# Innovative Data Science to Transform Health Care: All the Pieces Matter

**DOI:** 10.5334/egems.314

**Published:** 2019-08-28

**Authors:** Andrew L. Masica, José J. Escarce

**Affiliations:** 1 Center for Clinical Effectiveness, Baylor Scott and White Health, Dallas, TX, US; 2 Division of General Internal Medicine and Health Services Research, Department of Medicine, David Geffen School of Medicine at UCLA, Los Angeles, CA, US; 3 Department of Health Policy and Management, UCLA Fielding School of Public Health, Los Angeles, CA, US

**Keywords:** Data Science, Learning Health Systems, Electronic Health Records, Health Services Research, Analytics

## Abstract

This issue of eGEMS focuses on application of data science as a driver of health care transformation. Importantly, quantitative or qualitative analysis with a particular method is only one downstream step in the process of leveraging data. Effective analytics occurs on a continuum with multiple complementary phases, categorized here as data acquisition, ensuring or enhancing data access and usability, data analysis, and dissemination. Each of these activities is encompassed in the series of papers presented.

Converging demographic, epidemiologic, and economic trends have created an imperative to accelerate health care transformation efforts in the United States. America is graying, with the 65-and-older population projected to nearly double in size in the next several decades, from 49 million today to 95 million in 2060 [1]. The nation’s chronic disease burden is also growing. Six in ten American adults have at least one chronic disease; these illnesses are leading causes of death and disability [2]. Significant health inequities are present in the U.S., as income, education level, and geographic location are key determinants of access to care and health status [3,4,5]. Driven by technological change, including both the development of new diagnostic and treatment interventions and the expansion of existing modalities to new patients, as well as by a confluence of other issues such as the spread of health insurance and system inefficiencies, health care costs are high, currently accounting for nearly one-fifth of the U.S. Gross Domestic Product [6,7,8,9]. Within this context, consensus exists that health care redesign initiatives should move the U.S. towards a more patient-centered system that consistently delivers high value.

Although there is still equipoise regarding selection and deployment of specific, scalable models to transform health care, the field of data science (methods, processes, and systems for leveraging data), is a common thread in achieving that goal. The health care sector has historically generated extensive amounts of data through numerous sources. Data volume has proliferated greatly over the past decade with widespread use of electronic health records (EHRs) and the broader digitalization of the health care industry. However, in isolation, data accrual is an insufficient end point to guide innovation and foster change. Acquired data must also be usable as substrate in a formative event-conversion of data into knowledge and actionable insights through analytics.

Methodologies for developing and analyzing very large, complex health care datasets are of increasing interest to both the health care operations and research communities. Although at an early stage of application in health care relative to other industries, these “big data analytics” approaches appear to be evolving into a promising way to improve outcomes and reduce costs [10]. Reflecting the critical nature of timely, reliable data and analytics to policy decision makers, the Agency for Healthcare Research and Quality (AHRQ) recently proposed a strategic initiative in value that builds off its core competencies in data and analytics [11]. Some salient tactics within the initiative include establishment of an integrated data, analytics, and information platform capable of providing a 360-degree view of the health care system, as well as exploring recruitment of the agency’s first Chief Data Officer. Likewise, in a 2018 report, the National Academy of Medicine noted that continued advancement in health care, “will depend substantially on improving the national data infrastructure and leveraging routinely collected data” [12].

The learning health system model is an example of a transformational framework underpinned by data science. The Institute of Medicine’s vision for an adaptable, learning health system of the future is described as, “a system in which science, informatics, incentives, and culture are aligned for continuous improvement and innovation, with best practices seamlessly embedded in the care process, patients and families active participants in all elements, and new knowledge captured as an integral by-product of the care experience” [13]. The foundations of a learning health system are thus data aggregation and subsequent translation of that information into knowledge, which drives a cycle of continuous improvement. The data leading to actionable insights can be derived both from day-to-day processes (e.g., clinic visits, billing) and specialized activities (e.g., quality improvement, research).

This issue of eGEMs focuses on innovative data sourcing, data management, or data use approaches representing data science strategies that can be applied in health care transformation. Importantly, quantitative or qualitative analysis with a particular method is only one downstream step in the process of leveraging data. Effective analytics occurs on a continuum with multiple complementary phases, categorized here as data sourcing and acquisition, ensuring or enhancing data access and usability, data analysis, application, and data sharing/dissemination. Each of these activities is encompassed in the series of papers presented.

## Data Sourcing and Acquisition

Observations garnered from a data set are a direct function of its source. Much of the data used in health care analytics stems from secondary sources or is collected for other purposes outside of generating new insights (such as a billing record, which exists mainly for transactional reasons). Although these types of data sets have advantages in terms of availability and lower acquisition cost, in many scenarios, the information is not granular enough to guide targeted improvement efforts. A good example of this limitation are the publicly reported data on the CMS Hospital Compare website [14]. Using 30-day hospital readmissions as a demonstrative case, benchmarked facility level performance on this metric is readily accessible; facilities and patients know where an individual hospital stands. However, success factors driving a good performance or barriers contributing to a bad performance on 30-day readmissions cannot be elucidated from the Hospital Compare data set alone. That determination requires a “deeper dive” looking into primary, internal data sources for unique patient characteristics, event trends, or care processes impacting readmission rates.

Two articles in this issue indicate the incremental value of obtaining primary data in the interests of better understanding care delivery. Ridgely et al. collected detailed information on eight health systems through informant interviews, surveys, and document review. The supplemental data revealed substantial variability in terms of structure and mechanisms used to influence physician practice. These key attributes of health system performance could not be discerned from secondary sources alone. Savitz et al. assessed health literacy as a communication barrier in treatment planning conversations for stable angina through use of a questionnaire given directly to patients. Inadequate health literacy was associated with greater decisional conflict, but did not correlate with differential knowledge levels. Interestingly, gaps were found in engagement and knowledge surrounding management for stable angina at all levels of health literacy, identifying an opportunity for care improvement interventions. In both papers, drilling down into primary data revealed new findings the authors would not have gleaned otherwise from secondary sources.

## Enhancing Data Access and Usability

Three papers in this issue discuss approaches to making policy-relevant data easier to analyze, interpret, and use. Bir et al. discusses an innovative approach to presenting data from a large-scale federal evaluation developed to make the results of the evaluation more useful. The approach consists of an interactive dashboard that synthesizes quantitative and qualitative information and allows users to access specific evidence elements of interest in a timely, convenient fashion. Stoto et al. discusses limitations of the data available to nonprofit hospitals when they work with community organizations to prepare Community Health Needs Assessments and program implementation strategies. However, the article also describes how exemplary community health improvement processes can be developed working within the constraints of the available data. Immergluck et al. conducts a geospatial analysis of community-acquired methicillin-resistant Staphylococcus aureus infections, which are associated with health disparities. The authors demonstrate how this type of analysis can identify significant “hotspots” for infection, suggesting place-based risk factors and facilitating surveillance and prevention. This set of manuscripts indicates the enormous potential of innovative data science in promoting better policies.

## Data Analysis and Application

Two articles in this issue demonstrate direct clinical applications of data to improve quality. In Author et al., investigators from Kaiser Permanente Southern California analyzed physicians’ responses to a relaxation of therapeutic targets for HbA1c among older patients, intended to prevent adverse effects. Not surprisingly, the probability of medication intensification declined for older patients, but there were no spillover effects on younger patients, suggesting that physicians paid close attention to differential therapeutic targets for different age groups. In Author et al., researchers used data from electronic health records to develop a prediction model for the development of pressure ulcers in the intensive care unit whose performance surpassed that of existing prediction rules. The EHR-based model could be used to improve the allocation and timing of prophylactic interventions. Collectively, the two articles demonstrate how existing clinical data can be used to enhance care delivery and allocate resources more efficiently.

## Data Sharing and Dissemination

The final two articles highlight the need for viable mechanisms to aggregate and share data as a pathway to maintain data science’s position as a pillar for health care transformation. The confederated data network model, where multiple partners contribute similar data elements to a centralized location, is a solution that addresses the inefficiencies of stakeholders building collaborative databases on a case-by-case basis. Data networks have additional advantages in terms of size and diversity of information available. The PCORNet Common Data Model is just one of many successful examples of this frequently employed approach [15]. Morrato et al. summarize lessons learned in seeking the sustainability of the Scalable Architecture for Federated Translational Inquiries Network (SAFTINet), an electronic health data network involving over 50 primary care practices in three states. Using a commercialization readiness evaluation schema, SAFTINet customers identified data credibility, ease of use, and relevance to daily work as vital attributes of a sustainable network. Adams et al. describes how state-university partnerships can help states leverage their Medicaid data, build analytic capacity, and create evidence-based policy. The vehicle for these activities is the Medicaid Outcomes Distributed Research Network, a collaborative that supports multi-state analyses, uses a common data model across states, and increases the rigor of Medicaid policy evaluations.

Data science builds on a long tradition of health services research that has helped us to understand how our health care system functions and the ways in which health care and social factors influence the health of Americans. Nearly all of what we know about how our health care system and the key actors within it work—the functioning of health care markets; provider and patient responses to economic incentives and noneconomic arrangements; facilitators and barriers to access, including the importance of health insurance; the key role of technological change in driving health care cost growth; the ubiquitous disparities in health and health care; the gaps in quality; and innumerable other topics—results from the effort of dedicated and talented social scientists and health care providers who engage in health services research. Given the relentless pace of change in our health care system (as well as the societal variables that impinge upon it), expanding the scope and depth of health services research is vital. Delivery organizations, researchers, and policy makers continually identify new questions that require investigation and develop novel methods to enhance the validity and usefulness of their analyses. Data science is a welcome addition to the larger edifice of health services research and can only help to strengthen the field.

As illustrated in the articles contained within this issue of eGEMs, data science can be applied to improve clinical processes and quality of care, monitor and adjust program performance, and contribute to policy development. An essential currency of health system transformation towards better quality, higher value care is expedited acquisition of actionable knowledge. Ongoing investment in data science creates health care system capacity to successfully execute that activity.
